# Improving macromolecular atomic models at moderate resolution by automated iterative model building, statistical density modification and refinement

**DOI:** 10.1107/S0907444903009922

**Published:** 2003-06-27

**Authors:** Thomas C. Terwilliger

**Affiliations:** a Mail Stop M888, Los Alamos National Laboratory, Los Alamos, NM 87545, USA

**Keywords:** density modification, model building, refinement

## Abstract

An iterative process for improving the completeness and quality of atomic models automatically built at moderate resolution (up to about 2.8 Å) is described. The process consists of cycles of model building interspersed with cycles of refinement and combining phase information from the model with experimental phase information (if any) using statistical density modification. The process can lead to substantial improvements in both the accuracy and completeness of the model compared with a single cycle of model building. For eight test cases solved by MAD or SAD at resolutions ranging from 2.0 to 2.8 Å, the fraction of models built and assigned to sequence was 46–91% (mean of 65%) after the first cycle of building and refinement, and 78–­95% (mean of 87%) after 20 cycles. In an additional test case, an incorrect model of gene 5 protein (PDB code 2gn5; r.m.s.d. of main-chain atoms from the more recent refined structure 1vqb at 1.56 Å) was rebuilt using only structure-factor amplitude information at varying resolutions from 2.0 to 3.0 Å. Rebuilding was effective at resolutions up to about 2.5 Å. The resulting models had 60–­80% of the residues built and an r.m.s.d. of main-chain atoms from the refined structure of 0.20 to 0.62 Å. The algorithm is useful for building preliminary models of macromolecules suitable for an experienced crystallographer to extend, correct and fully refine.

## Abstract 2

A procedure for iterative model-building, statistical density modification and refinement at moderate resolution (up to about 2.8 Å) is described.

## 1. Introduction

Iterative model building and refinement has proven to be an exceptionally powerful tool for automatic interpretation of macromolecular electron-density maps where the diffraction data extend beyond about 2.3 Å (Lamzin & Wilson, 1993 ▶; Perrakis *et al.*, 1997 ▶, 1999 ▶, 2001 ▶; Morris *et al.*, 2002 ▶). In this approach, implemented in *ARP* (Lamzin & Wilson, 1993 ▶) and later in *wARP* (Perrakis *et al.*, 1999 ▶), electron density in a map is interpreted initially in terms of peaks corresponding to atomic coordinates. These ‘free atoms’ are subsequently refined and interpreted in terms of a macromolecular structure, which can be further refined. The refined model or models are then used to provide updated estimates of crystallographic phases, leading to a new electron-density map, and the process is repeated until no further improvements to the model occur.

The free-atom model-building approach works well when data is available to near-atomic resolution or better (<2.3 Å; Perrakis *et al.*, 1999 ▶), but is limited by the need to identify peaks of density at the positions of atomic coordinates. At lower resolution, atoms are not well defined in the electron density and the free-atom method of initiating model building has not been as useful, although related methods have been used to improve electron-density maps at resolutions up to 3 Å (Vellieux, 1998 ▶). Recently, several methods for automated model building at moderate resolution (<3 Å) have been developed. Each of these methods relies on features of macromolecular electron-density maps on a larger scale than individual atoms to begin model building. Oldfield (2002 ▶) described a method to identify helices and sheets and then extended these segments one amino acid at a time to trace a polypeptide. Levitt (2001 ▶) uses an interpretation of the connected regions of the map (the ‘bones’ of Greer, 1985 ▶) to identify helices and sheets and then also extends them to trace a polypeptide. Ioerger & Sacchettini (2002 ▶) used a pattern-matching approach to identify C^α^ positions and trace polypeptide backbones. We recently described another method (Terwilliger, 2001*a* ▶, 2003*a* ▶,*b* ▶) for identifying the locations of helices and sheets based on the template-convolution method of Cowtan (1998 ▶), followed by correlation-based refinement of the position and orientations of the templates and choosing a fragment of a helical or sheet region from a library constructed from refined protein structures. These helices and sheets are then extended using tripeptide fragments from a library constructed from a set of refined protein structures.

Here, we show that the quality and completeness of automatic model building at moderate resolution can be substantially improved by alternating model-building cycles with cycles of phase improvement. The phase improvement is carried out with statistical density modification (previously known as maximum-likelihood density modification; Terwilliger, 2000 ▶) and can include information based on the refined partial model, information from experiments and information from classical density-modification sources such as solvent flattening and non-crystallographic symmetry.

## 2. Methods

### 2.1. Initial phase calculations from SAD or MAD data

Initial phase calculations were carried out using statistical density modification with *RESOLVE* (Terwilliger, 2000 ▶) based on phase probability distributions obtained from SAD or MAD data using *SOLVE* (Terwilliger & Berendzen, 1999 ▶). Non-crystallographic symmetry (NCS) in the structures was identified from any NCS present in the heavy-atom sites and was verified by analysis of the correlation of density at NCS-related positions in the *SOLVE* electron-density map (Terwilliger, 2002*a* ▶,*b* ▶). NCS was used as a source of prior information about the electron-density map in much the same way as the flatness of the solvent region (Terwilliger, 2000 ▶, 2002*b* ▶). The statistical density-modified map and the NCS operations, if any, were used as the input to automated model building.

### 2.2. Model building

Automated model building was carried out as described previously (Terwilliger, 2003*a* ▶,*b* ▶). This procedure requires an electron-density map, the sequences of any protein chains and any non-crystallographic symmetry information that is available. It produces an atomic model consisting of linked fragments of polypeptide chain from fragment libraries and side chains from rotamer libraries.

### 2.3. Refinement

Restrained maximum-likelihood refinement was carried out with *REFMAC*5 (version 5.1.24; Murshudov *et al.*, 1997 ▶) and default parameters for a poor low-resolution model, except that no scaling of reliability of phases was performed. Phase information from the current best phase set was included in refinement. Overall thermal factor refinement was used with tight restraints (Wmat = 0.15) and damping of shifts was included (Pdamp = 0.5, Bdamp = 0.5). A bulk-solvent model was included with Bbulk = 200 and SCbulk = −0.05. It should be noted that these parameters were not optimized and that optimal values are likely to depend on the resolution of the data and the quality of the model. A total of 20 cycles of refinement were carried out for each application of *REFMAC*5. Reflections were divided randomly into a test set (5%) and a working set (95%) at the beginning of iterative refinement and the same test set was used throughout the process. Non-crystallographic symmetry restraints were not included in refinement; however, some model-based non-crystallographic symmetry information could be propagated through the image-based phasing procedure (which includes non-crystallographic symmetry), so there is a possibility that the free *R* factors for cases with non-crystallographic symmetry could be slightly biased. A user-defined test set can be read in using the *CCP*4 conventions (Collaborative Computational Project, Number 4, 1994 ▶) in order to reduce this potential non-crystallographic symmetry bias (Kleywegt, 1996 ▶).

### 2.4. Estimation of electron density based on one model

Electron density was calculated from unrefined or partially refined models in two steps. Firstly, electron density was calculated directly from the model for all points within the distance rad_max of an atom, where rad_max corresponds to the resolution of the data or 2.5 Å, whichever is larger. The electron density calculated in this way is therefore only defined at points near to atoms. An overall thermal factor and an incremental thermal factor for side-chain atoms (depending on the number of bonds between the atom and C^α^) were then estimated by maximizing the correlation of the calculated electron density with the density in the current best electron-density map. In cases where no prior electron-density map exists, these parameters were not optimized.

### 2.5. Estimation of electron density based on several non-independent models

To combine estimates of electron density from several atomic models, a real-space procedure related to the reciprocal-space weighting procedure of Perrakis *et al.* (1997 ▶) was used. The potential advantage of a real-space averaging method is that two models that cover partially overlapping regions of the asymmetric unit can be combined in different ways in the regions where they overlap and the regions where only one model has density. Two methods were used to combine electron density from multiple models. In the first (unweighted average) method, the electron density at each point was the simple average of the electron-density values for all models that have density defined at that point. In the second (weighted average) method, the covariances of the electron densities for each pair of the various models were calculated in the regions where both members of each pair are defined. This covariance matrix was then used to calculate a minimum-variance estimate of the electron density as described by Read (2001 ▶). This calculation requires estimates of the correlations between each electron-density map and the true map. These correlations and their overall average cc_avg_ were estimated as the mean correlations of *F*_obs_ with *F*_calc_, estimated in shells of resolution. Although the map correlation and the structure-factor amplitude correlation are not expected to be equal, they have the same range (−1 to 1), similar values and similar trends (increasing values with increasing quality of the model), which is sufficient for the present purpose. In cases where the covariance matrix was singular or any weights on any electron-density maps were negative, the map with the most negative weight was removed and the calculation was repeated. For all points where electron density from some models was not defined, the weights on the remaining models were increased to yield the same sum of weights. The two methods of estimation of electron density based on several models were generally both used during a set of cycles of model building and phase recombination, with the weighted average method being used on most cycles and the unweighted average method used every fifth cycle.

### 2.6. Statistical density modification with an electron-density target for part of the asymmetric unit (image-based phase estimation)

Information about the electron density in part of the asymmetric unit was used as a source of phase information in statistical density modification in the same way as information about solvent flatness or NCS symmetry. For each of these sources of information, an estimate of the probability distribution for possible values of electron density at each point in the map is needed. For the overall distributions of density in the solvent- and macromolecule-containing regions, these distributions have been described (Terwilliger, 2000 ▶) and consist of fits of distributions for solvent and protein regions calculated from model data, broadened by Gaussian functions. For NCS-related points in the map, the distributions are modeled by a single Gaussian with a width based on the r.m.s. difference between densities at NCS-related points (Terwilliger, 2002*b* ▶). For the calculated electron-density map, the distributions were also modeled by a single Gaussian function. Model density was scaled to the density in the current best electron-density map (if any) and used as the target electron density. The uncertainty in the target electron-density values σ was calculated from the estimates made above of the mean correlation cc_avg_ of the model and true electron density and the r.m.s. value of the current electron-density map, ρ_r.m.s._, using the approximate relation σ = ρ_r.m.s._(1 − cc_avg_^2^)^1/2^. If no electron-density map was available, then the r.m.s. value of the model electron-density map was used in this relation instead. Once probability distributions for electron density at each point in the asymmetric unit are defined, the map probability function (previously known as the map likelihood function; Terwilliger, 2001*b* ▶) can be used to estimate phase probabilities from this information alone or in combination with prior phase information.

### 2.7. Iterative phase combination using statistical density modification

Phase combination by statistical density modification was carried out iteratively. For each iteration, the electron-density map produced in the previous iteration (or a starting density-modified experimental map) was used as the starting electron-density map for density modification. Any prior phase probability information and the starting values of NCS operators used were identical to those used in the initial statistical density-modification calculation. The probability that each point was in the solvent was recalculated after each iteration using the starting electron-density map. In this process, the calculated electron density from the model was the principal source of information about the expected map density that varied from iteration to iteration of the model-building and density-modification process. Three cycles of density modification were carried out during each iteration of statistical density modification. Additional cycles had little effect because all the sources of information about expected values of density in the map were constant during a given iteration and the statistical density-modification procedure converged rapidly. Once density modification was complete, a new map was calculated and the process was repeated.

### 2.8. Cross-validated statistical density modification with information from a model (omit prime-and-switch phasing)

A reduced-bias electron-density map was calculated from an atomic model in two steps. Firstly, target electron density was estimated from the model as described above and one cycle of image-based phase estimation was carried out to yield a starting set of phases and figures of merit. Next, the asymmetric unit was divided into approximately 20 omit regions. In each cycle of cross-validation, prime-and-switch phasing was carried out as described previously (Terwilliger, 2001*b* ▶) beginning with the image-based starting set of phases, but additionally including the target electron-density map based on the model for all points except those in one omit region (Shah *et al.*, 1997 ▶). Three cycles of prime-and-switch phasing with the omit electron-density target were carried out as part of each cycle of cross-validation, yielding an ‘omit’ electron-density map defined in the region where model electron density was not included. The omit regions from all the cycles of cross-validation were then combined to create a composite ‘omit prime-and-switch’ electron-density map.

### 2.9. Combination of model building and model refinement

Model building and refinement were combined in one of two ways: a simple alternation of model building and refinement and a multi-step procedure of model building, refinement, model extension and side-chain refitting. In the multi-step procedure, a model is built into an electron-density map as described previously (Terwilliger, 2003*a* ▶,*b* ▶). The model is then refined and the refined model is used as a starting point for a model-rebuilding step. In the rebuilding step, chains in the refined model are trimmed back to match electron density in the current map and are then extended using tripeptide-fragment libraries in the same way as during initial model building (Terwilliger, 2003*a* ▶). The side chains are identified in the same fashion (Terwilliger, 2003*b* ▶), except that now the definition of the side-chain orientation is based on a refined model, not the initial model. In the multi-step procedure this refinement, extension and side-chain refitting process was carried out twice. In each iteration of the whole process the model was rebuilt, but fragments of the model from the previous iteration were used as starting points for rebuilding in addition to any helix or strand positions found in the FFT-based pattern-matching process used for initial model building (Terwilliger, 2003*a* ▶).

## 3. Results and discussion

The key step in this iterative model-building, density-modification and refinement procedure is to use electron density from a refined model as a source of information for statistical density modification. The *ARP*/*wARP* procedure (Perrakis *et al.*, 1999 ▶) has demonstrated clearly that a model can be built and refined with some accuracy beginning with a map that has a significant level of noise and that the electron density calculated from such a model can be more accurate (in the region occupied by the model) than the original map. The novel aspects of the present method are the use of a model-building procedure that is effective at moderate resolution (Terwilliger, 2003*a* ▶,*b* ▶) and the use of statistical density modification in the phase-recombination step of iterative model building and refinement.

### 3.1. Iterative model building, density modification and refinement with experimental phase information

**Figure 1:**
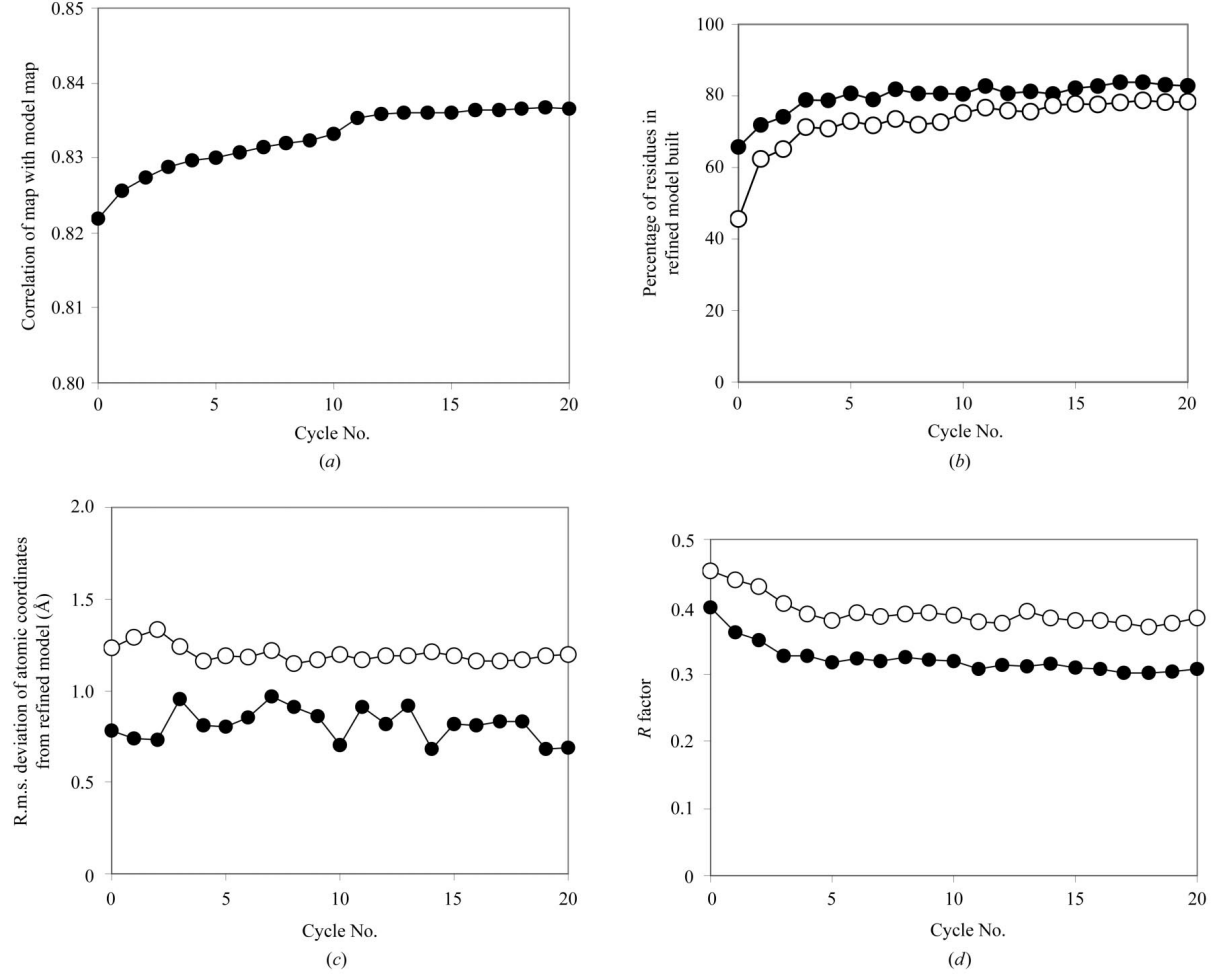
Iterative model building of UTP synthase (Gordon *et al.*, 2001 ▶) at 2.8 Å. (*a*) Correlation of statistical density-modified map with map calculated from reference refined model of UTP synthase (Gordon *et al.*, 2001 ▶) at the end of each cycle. (*b*) Percentage of main-chain atoms (filled circles) and side-chain atoms (open circles) built in each cycle. (*c*) R.m.s. coordinate difference between models built in each cycle with the reference refined model for main-chain atoms (filled circles) and side-chain atoms (open circles). (*d*) Working *R* factor (filled circles) and free *R* factor (open circles) at the end of each cycle.

Fig. 1 ▶ shows the progress of iterative model building in the case of SAD data from UTP synthase at a resolution of 2.8 Å (Gordon *et al.*, 2001 ▶). To evaluate the quality of models built by this procedure, the model of UTP synthase refined at a resolution of 2.0 Å (PDB code 1e8c) was used as a reference. Fig. 1 ▶(*a*) shows the correlation of the density-modified map at the beginning of each cycle with the map based on the reference model. On the zeroth cycle this density-modified map is that produced by statistical density modification without using model information (Terwilliger, 2000 ▶) and for this UTP synthase SAD data the starting correlation was 0.822. Over the course of 20 cycles of model building, this correlation gradually increased to 0.837. Each of these cycles consisted of density modification using electron density from the current model, model building, refinement of the model and two cycles of chain extension and refinement. In Figs. 1 ▶(*b*)–1(*d*), the characteristics of the refined models at the end of each cycle are shown. As in Fig. 1 ▶(*a*), the zeroth cycle corresponds to the model built and refined on the basis of the initial density-modified map. In this zeroth cycle of model building, 71% of main-chain residues and 52% of the corresponding side chains were built. By the end of 20 cycles, 79% of the both main-chain residues and side chains were built. The overall accuracy of atomic coordinates improved slightly during the course of model building. In the zeroth cycle the r.m.s. difference in position between main-chain atom coordinates in the model built by the present procedure and those in the refined reference model was 0.78 Å; after 20 cycles it was reduced to 0.69 Å. The working *R* factor (at 2.8 Å) of the models decreased from 0.40 at the end of refinement of the initial model in the zeroth cycle to 0.31 in the 20th cycle. The corresponding free *R* factors decreased from 0.45 to 0.38 (however, there could be a slight bias in these free *R* factors as the twofold symmetry of UTP synthase was used in the density-modification steps).

**Figure 2:**
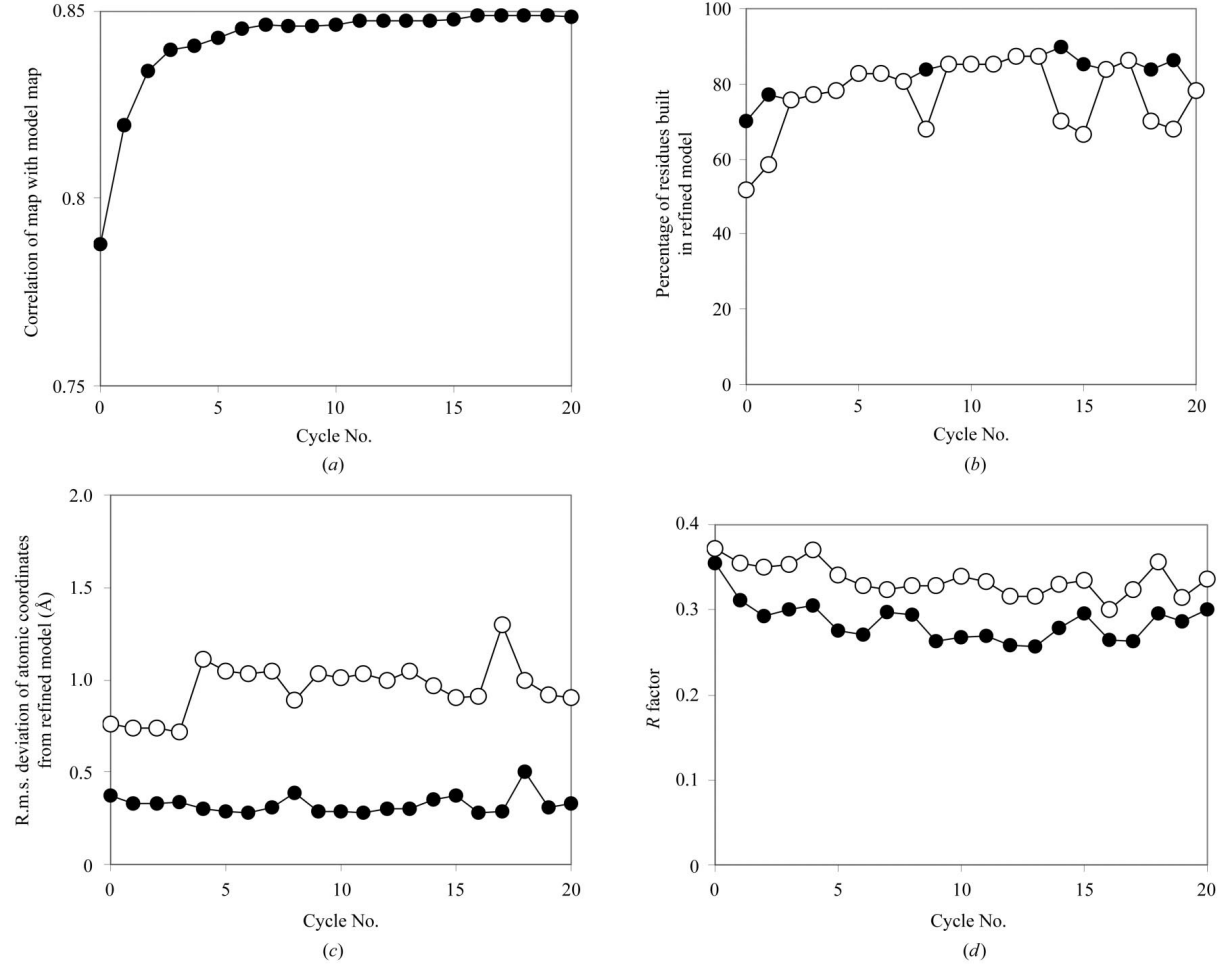
Iterative model building of gene 5 protein (Skinner *et al.*, 1994 ▶) at 2.6 Å. (*a*)–(*d*) as in Fig. 1 ▶.

**Figure 3:**
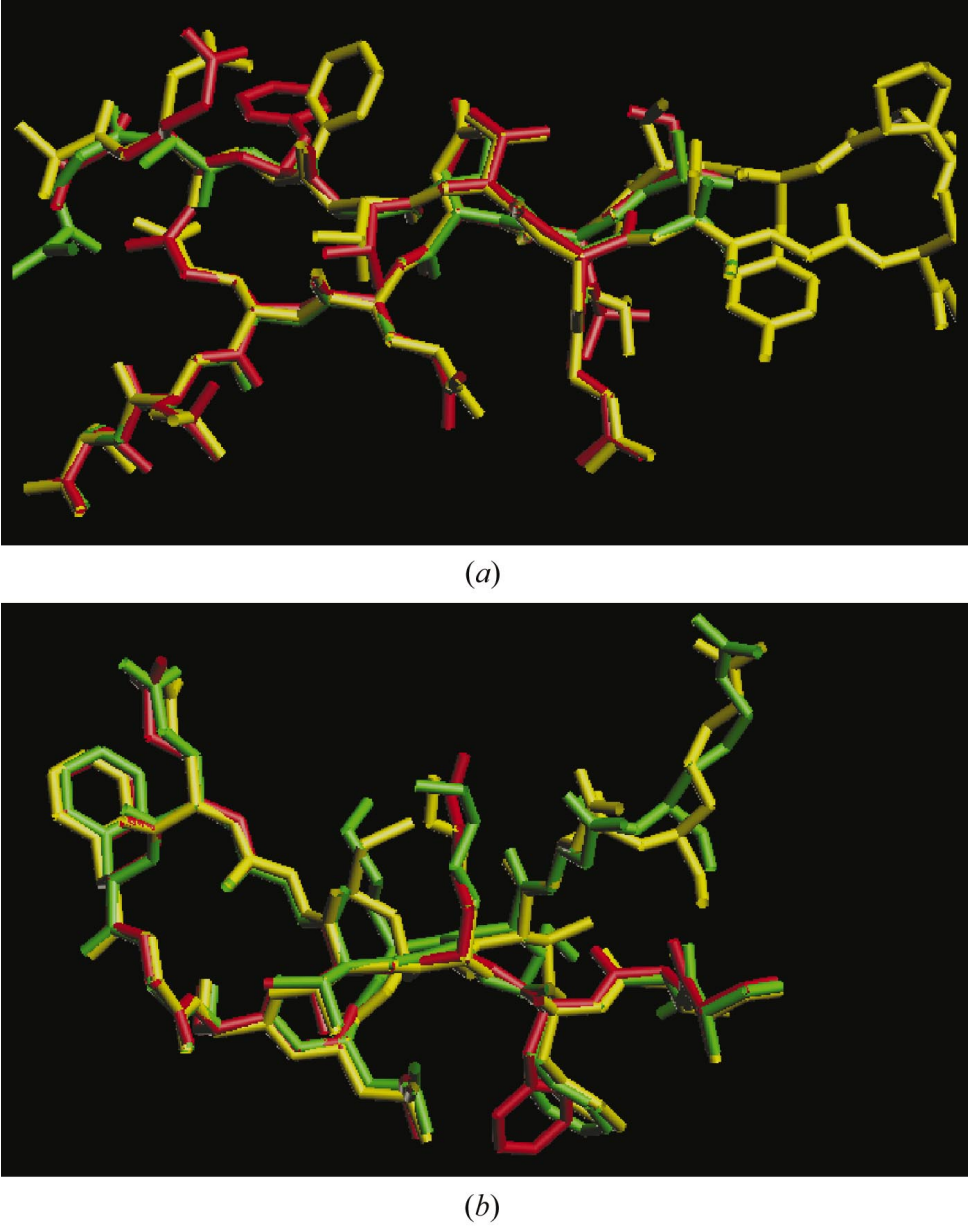
Segments of gene 5 protein models built automatically. (*a*) Residues 11–33. (*b*) Residues 66–80. In each case the refined model is in yellow, the model after one cycle of building is in red and the model after 20 cycles of building is in green. Figures constructed with *O* version 8.0 (Jones *et al.*, 1991 ▶).

Fig. 2 ▶ shows the benefit of iterative model building in the case of slightly higher resolution (2.6 Å) data from gene 5 protein (Skinner *et al.*, 1994 ▶). The reference model was PDB entry 1vqb, refined at 1.8 Å (Skinner *et al.*, 1994 ▶). The correlation of the density-modified maps with the map based on the reference model improved very substantially from 0.79 to 0.85 during the course of iterative model building in this case. Automatic model building was able to place 79% of the main-chain residues and 52% of side chains in the first cycle and 79% of both main chain and side chains in the 20th cycle (Fig. 2 ▶ *b*). The r.m.s. difference between main-chain atoms and the refined coordinates of gene 5 protein (Skinner *et al.*, 1994 ▶) decreased from about 0.37 to 0.33 Å during the iterative model building (Fig. 2 ▶ *c*) and for side-chain atoms it became slightly worse overall, increasing from 0.76 to 0.90 Å. The working *R* factor at 2.6 Å decreased from 0.36 to 0.30 during the course of iterative refinement and model building and the free *R* factor decreased from 0.37 to 0.34. Fig. 3 ▶ illustrates representative sections of the refined model (in yellow), the model after one cycle of building (red) and the model after 20 cycles of building (green).

Table 1 ▶ summarizes iterative model building results for eight proteins, including the UTP synthase and gene 5 protein cases shown in Figs. 1 ▶ and 2 ▶. In all eight cases, the iteration of model building resulted in a substantially more complete and more accurate model than was obtainable in the first cycle of model building. Overall, the fraction of the models built and assigned to sequence was 46–91% (mean of 65%) after the first cycle of building and refinement, and 78–95% (mean of 87%) after 20 cycles.

The preceding examples show that iterative statistical density modification, model building and refinement can be useful in improving the completeness of atomic models at moderate resolution (at least up to about 2.8 Å) in cases where a starting set of experimental phase probability estimates is available. The experimental phase probabilities are very useful in this procedure because they can be combined with model-based information during every cycle of the process and often contribute as much or more to the phase information as the model.

### 3.2. Iterative model building, density modification and refinement without experimental phase information

A more difficult problem is that of iterative model-building when no experimental phase probability distributions are available, such as in the case of rebuilding models in molecular replacement (Rossmann, 1972 ▶). The iterative model-building and refinement process carried out by *ARP*/*wARP* (Perrakis *et al.*, 1999 ▶) has been very successful in this application (Perrakis *et al.*, 2001 ▶). In addition to the absence of experimental phase information in this case, model bias arising from the starting model can exist. To reduce model bias, we use a variation on our method of ‘prime-and-switch’ phasing (Terwilliger, 2001*b* ▶) to calculate a reduced-bias initial electron-density map. In the method described earlier (Terwilliger, 2001*b* ▶), a starting set of phases is calculated from a model and then in an iterative process phases are estimated by maximizing the agreement of the features of the map with expectations (*e.g.* a flat solvent or the presence of NCS), without reference to the starting set of phases. In the variation used here, a similar process is carried out but using some additional information and an ‘omit’ procedure, as described above. For each cycle, several ‘omit’ sub-cycles are carried out. In each sub-cycle, a calculated electron-density map is included as the information for image-based phasing (see §[Sec sec2]2) for all points in the asymmetric unit outside of an ‘omit’ region. The omitted regions for all the sub-cycles are then combined to form a composite electron-density map.

We used the gene 5 protein structure to test the application of iterative model building, density modification and refinement to a case of model rebuilding. The structure of gene 5 protein has been determined several times by X-ray crystallographic methods (McPherson *et al.*, 1979 ▶; Brayer & McPherson, 1983 ▶; Skinner *et al.*, 1994 ▶). The two more recent determinations were carried out using crystals of gene 5 protein in the same space group *C2* crystal form, first by MIR methods (Brayer & McPherson, 1983 ▶) and later by MAD phasing (Skinner *et al.*, 1994 ▶). We take the structure of Skinner *et al.* (1994 ▶) (PDB code 1vqb) as our reference in this analysis because it is at the higher resolution of these structures (1.8 Å); it has subsequently been refined at even higher resolution (1.6 Å; S. Su, Y.-G. Gao, H. Zhang, T. C. Terwilliger & A. H.-J. Wang, unpublished results; PDB code 1gvp) and it is very similar to a structure built on the basis of NMR data (Folkers *et al.*, 1994 ▶). The structure of Brayer & McPherson (1983 ▶) (PDB code 2gn5) was determined at the moderate resolution of 2.3 Å and differs from the higher resolution structure 1vqb in the loops and in the register of the β-strands. The overall r.m.s. difference between corresponding protein atoms in 2gn5 and 1vqb is 1.75 Å for main-chain atoms and 3.53 Å for side-chain atoms.

**Figure 4:**
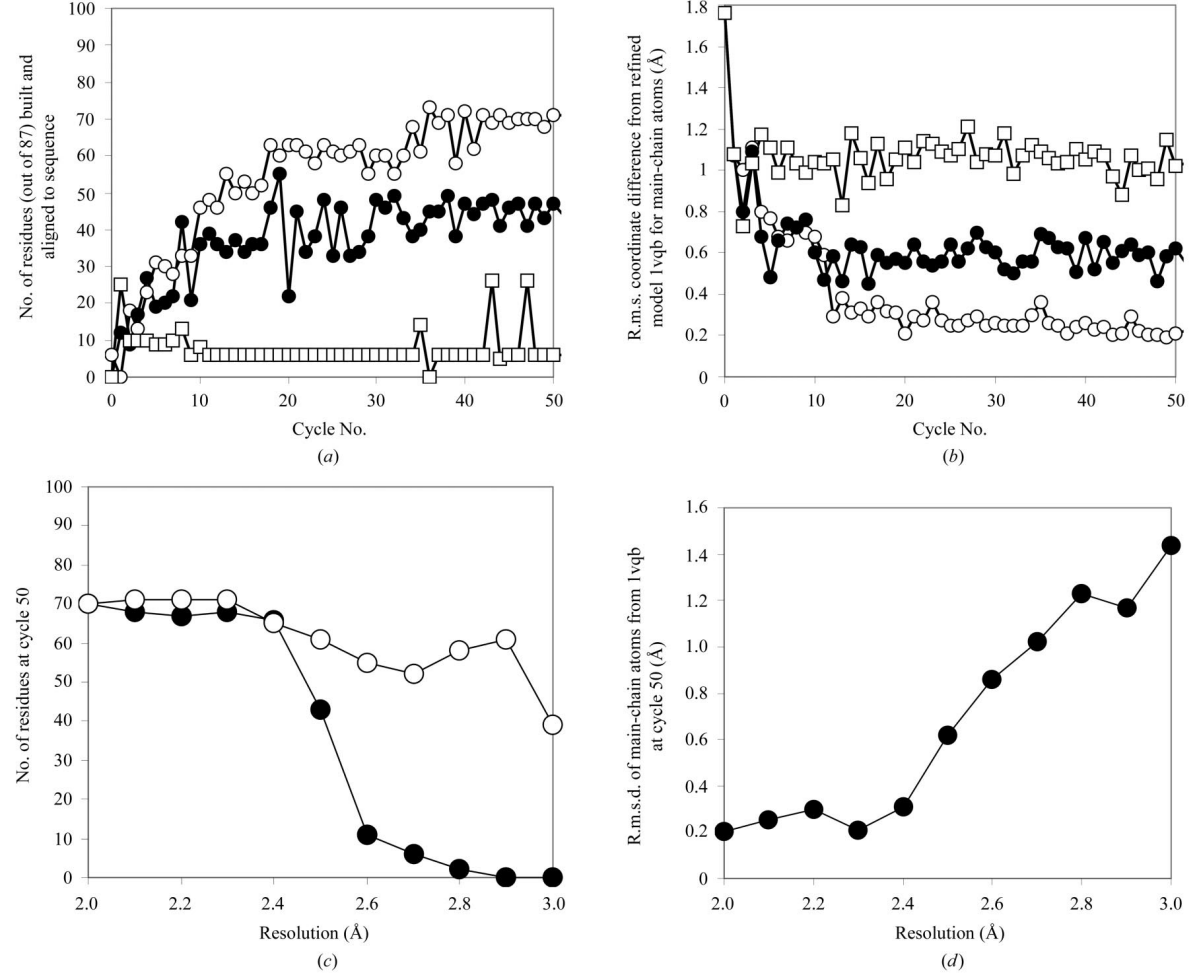
Iterative rebuilding of gene 5 protein beginning with structure 2gn5 and using structure-factor amplitudes corresponding to 1vqb. (*a*) Number of residues built and aligned to sequence as a function of cycle number and resolution of data used (open circles, 2.3 Å; closed circles, 2.5 Å; open squares, 2.7 Å). Gene 5 protein has 87 amino-acid residues; the refined model 1vqb contains 86. (*b*) R.m.s. coordinate difference between refined intermediate models and 1vqb for main-chain atoms (symbols as in *a*). (*c*) Number of residues built in 50 cycles as a function of resolution of data used. Open circles, main chain; closed circles, side chains. (*d*) R.m.s. coordinate difference between refined intermediate models at cycle 50 as a function of the resolution of the data used.

We used the structure 2gn5 as a starting point for iterative model building, density modification and refinement. In this procedure, the structure-factor amplitudes used were those measured from the *C*2 crystal form of gene 5 protein and which had been used as the basis for refinement of the 1vqb structure (Skinner *et al.*, 1994 ▶). These structure-factor amplitudes were measured to a resolution of 1.8 Å. For the present purpose, data at varying resolutions were used to assess the utility of the method. Fig. 4 ▶(*a*) shows the number of residues built and assigned to sequence using data to 2.3, 2.5 and 2.7 Å. Using data to 2.3 Å, 70 of the 87 residues in gene 5 protein could be built and side chains could be built and correctly assigned to the sequence for all of them. The total number of residues built (whether side chains were built or not) increased from 46 in the first cycle (with six side chains built and assigned to sequence) to 70 in the 50th cycle (with all assigned to sequence). At a resolution of 2.5 Å, 61 residues could be built in 50 cycles, of which 47 residues could be assigned to the sequence. At 2.7 Å, 52 residues could be built in 50 cycles, but just six residues could be assigned to the sequence.

Fig. 4 ▶(*b*) shows the r.m.s. coordinate difference between partially refined intermediate models built using data to 2.3, 2.5 and 2.7 Å and the corresponding atoms in the reference model 1vqb (Skinner *et al.*, 1994 ▶). At a resolution of 2.3 Å, the r.m.s. coordinate difference decreases from 1.75 Å (for the starting model) to just 0.2 Å over the course of 50 cycles. At resolutions of 2.5 and 2.7 Å the coordinate differences are somewhat higher: 0.62 and 1.02 Å, respectively.

Fig. 4 ▶(*c*) shows the number of residues built as a function of resolution as well as the number of side chains placed in the corresponding models, while Fig. 4 ▶(*d*) shows the corresponding main-chain coordinate differences from the reference model 1vqb. At resolutions of about 2.5 Å or better, the iterative algorithm is capable of building much of the main chain (61 or more of 87) and side chains (43 or more of 87) and the r.m.s. coordinate difference between these models and the reference model 1vqb is 0.6 Å or less.

### 3.3. Basis for model improvement through iterative model building, density modification and refinement

There are several reasons why iterative cycles of model building and density modification might be expected to improve the overall completeness and accuracy of the model produced. The most obvious one, and the principal reason for applying the method, is that the map used for model building can be more accurate after inclusion of phase information from the partial model. Over the course of iterative model building, the model contains a larger number of atoms and the resulting phase information improves. While this seems likely to be the major contribution to the utility of the method, it may not be the only important factor because the extent of phase improvement is relatively small (on average, an increase in the effective figure of merit of 0.015 over the course of iterations in the eight test cases). A possible additional mechanism whereby a small improvement in the map could lead to a large improvement in the overall completeness of model building is that the inclusion of the refinement step leads iteratively to improved side-chain placement. Side-chain atom placement is dependent on the main-chain atoms in this procedure, as the side chains are identified and placed by superimposing templates for side-chain rotamers on the map using the coordinates of main-chain N, Ca and C atoms. Consequently, it seems possible that part of the large improvement in the quantity of side-chain atoms placed is owing to the refinement of main-chain atomic positions.

### 3.4. Other algorithms for iterative model building at moderate resolution

The procedures described here were carried out with statistical density-modification procedures (Terwilliger, 2000 ▶) and with an automatic model-building procedure (Terwilliger, 2003*a* ▶,*b*) based on placing fragments from a library built from refined protein structures. The approach is not specific to these particular methods, however. Other means of phase combination such as σ_*A*_-weighted phase recombination (Read, 2001 ▶) and other model-building procedures such as those of Ioerger & Sacchettini (2002 ▶), Levitt (2001 ▶) or Oldfield (2002 ▶) that can function at moderate resolution and procedures that include atomic refinement could also potentially yield improvement with an iterative approach.

### 3.5. Limitations of the method

The algorithm described here is useful for building a preliminary model, but is not suitable in its current form for fully automatic model building because it does not build a complete model and it does not fully check the model it builds for consistency with known features of macromolecules. At present, only features in its database are recognized; unusual amino acids, ligands, water molecules and nucleic acids are not yet in the databases used. The model-building software performs rudimentary checks for overlap of atomic positions (Terwilliger, 2003*a* ▶) and nearly all the model building is carried out with templates from refined protein structures, but the algorithm does not currently include a systematic check of conformations or van der Waals contacts. An additional limitation is that non-crystallographic symmetry restraints are currently not applied during the refinement process. It is likely that considerably improved models could be obtained by including them. Owing to these limitations, the current algorithm can provide an experienced crystallographer with a very good starting point for final model building and refinement but not with a final model.

## 4. Conclusions

Iterative model building and phase combination is found to yield considerably more accurate and more complete models than simply building a model into an electron-density map for cases where phase information is available at moderate resolution (<2.8 Å). The use of automated model-building algorithms capable of building models at moderate resolution has therefore extended the range of applicability of iterative model building and refinement (Perrakis *et al.*, 1999 ▶) up to about 2.8 Å. The procedures described here have been implemented in version 2.03 of *RESOLVE* and are available from http://solve.lanl.gov.

## Figures and Tables

**Table 1:** Test structures built using iterative model building and refinement

Structure	UTP synthase (Gordon *et al.*, 2001 ▶)	β-Catenin (Huber *et al.*, 1997 ▶)	2-Aminoethylphosphonate (AEP) transaminase (Chen *et al.*, 2002 ▶)	Gene 5 protein (Skinner *et al.*, 1994 ▶)	Hypothetical (*P. aerophilum* ORF; NCBI accession No. AAL64711; Fitz-Gibbon *et al.*, 2002 ▶)	NDP kinase (Pédelacq *et al.*, 2002 ▶)	Initiation factor 5A (Peat *et al.*, 1998 ▶)	Red fluorescent protein (Yarbrough *et al.*, 2001 ▶)
Resolution (Å)	2.8	2.7	2.6	2.6	2.6	2.4	2.1	2.0
Type of experiment	SAD	MAD	SAD	MAD	MAD	MAD	MAD	MAD
Figure of merit at start of model building 〈*m*〉	0.73	0.72	0.84	0.62	0.58	0.56	0.85	0.91
Residues in reference refined model†	1012 (2 × 506)	455	2232 (6 × 372)	86	494 (2 × 247)	556 (3 × 186)	136	936 (4 × 234)
% of main-chain built								
Cycle 1	66	81	92	71	86	59	81	90
Cycle 20	83	95	94	79	95	85	85	91
% of side chains built								
Cycle 1	46	64	91	52	85	53	81	50
Cycle 20	78	86	93	79	95	85	85	91
R.m.s. coordinate difference‡								
Main chain	0.69	0.92	0.48	0.33	0.26	0.31	0.21	0.33
Side chain	1.2	1.25	1.09	0.9	1.14	1.12	0.87	1.16
Change in map correlation with map based on reference refined model† from beginning to 20th cycle	0.015	0.009	0.002	0.061	0.010	0.012	0.003	0.003
Working *R* factor								
Cycle 1	0.40	0.35	0.27	0.36	0.30	0.39	0.33	0.34
Cycle 20	0.31	0.27	0.26	0.30	0.27	0.28	0.31	0.28
Free *R* factor								
Cycle 1	0.45	0.39	0.30	0.37	0.35	0.42	0.33	0.36
Cycle 20	0.38	0.31	0.30	0.34	0.32	0.33	0.32	0.31

† The reference refined model in each case is either the deposited PDB entry for this structure or the unpublished refined structure, in each case built without using *RESOLVE* model building.

‡ R.m.s. coordinate difference between model at the 20th cycle and reference refined model (Å).
